# MucR binds multiple target sites in the promoter of its own gene and is a heat‐stable protein: Is MucR a H‐NS‐like protein?

**DOI:** 10.1002/2211-5463.12411

**Published:** 2018-03-31

**Authors:** Ilaria Baglivo, Luciano Pirone, Gaetano Malgieri, Roberto Fattorusso, Roy Martin Roop II, Emilia Maria Pedone, Paolo Vincenzo Pedone

**Affiliations:** ^1^ Department of Environmental, Biological and Pharmaceutical Sciences and Technologies University of Campania ‘Luigi Vanvitelli’ Caserta Italy; ^2^ Institute of Biostructures and Bioimaging C.N.R. Naples Italy; ^3^ Department of Microbiology and Immunology Brody School of Medicine East Carolina University Greenville NC USA

**Keywords:** *Brucella*, H‐NS, infectious disease, MucR, virulence, zinc‐finger protein

## Abstract

The protein MucR from *Brucella* spp. is involved in the expression regulation of genes necessary for host interaction and infection. MucR is a member of the Ros/MucR family, which comprises prokaryotic zinc‐finger proteins and includes Ros from *Agrobacterium tumefaciens* and the Ml proteins from *Mesorhizobium loti*. MucR from *Brucella* spp. can regulate the expression of virulence genes and repress its own gene expression. Despite the well‐known role played by MucR in the repression of its own gene, no target sequence has yet been identified in the *mucR* promoter gene. In this study, we provide the first evidence that MucR from *Brucella abortus* binds more than one target site in the promoter region of its own gene, suggesting a molecular mechanism by which this protein represses its own expression. Furthermore, a circular dichroism analysis reveals that MucR is a heat‐stable protein. Overall, the results of this study suggest that MucR might resemble a H‐NS protein.

**Abbreviations**
CD: circular dichroism
EMSA: electrophoretic mobility shift assay
*T*_m_: temperature of melting

Brucellosis is the most widespread zoonosis in the world caused by *Brucella* spp., which are Gram‐negative α‐proteobacteria 1. Infectious abortion is caused by *Brucella abortus* and *Brucella melitensis* in cattle, sheep and goats, and it constitutes a serious threat to the livestock industry and a source of human infection 1 with an estimated annual incidence of 500 000 human cases worldwide 2. If untreated, brucellosis can develop into a chronic infection with musculoskeletal localization 1. For these reasons, the study of the molecules involved in regulation of *Brucella* virulence genes and the mechanism that *Brucella* adopts to control virulence gene expression are of considerable interest. The protein MucR from *B. abortus* and *B. melitensis* is involved in the expression regulation of genes necessary for host interaction and infection, and it is able to repress the expression of its own gene 3, 4. Mutations in the *mucR* gene of *B. melitensis* and *B. abortus* lead to attenuated *Brucella* strains 3, 4, 5. Conversely to the clear evidence of the role of MucR from *Brucella* in the repression of its own gene, no target sequence has yet to be identified in the *mucR* promoter region. MucR is a Ros/MucR protein family member and shares the conserved prokaryotic zinc‐finger domain with the other members of this protein family 6, 7, 8, 9, 10. The prokaryotic zinc‐finger domain is responsible for DNA‐binding of the protein Ros from *Agrobacterium tumefaciens* and the Ml proteins from *Mesorhizobium loti* 6, 7, 8, 9, 10, 11, 12, 13, 14, 15, 16, 17. In *A. tumefaciens*, Ros represses the virulence gene expression 18, while the biological role of Ml proteins in *M. loti* has not been yet clarified. However, the ability of Ml proteins to bind the promoter of the *exoY* gene and the expression of *ml* genes during stationary phase of planktonic growth or in biofilm suggest that the Ml proteins are involved in the regulation of exopolysaccharide biosynthesis 17 like MucR in *Sinorhizobium meliloti* 19 and MucR in *B. melitensis* 3.

Recently, using Electrophoretic Mobility Shift Assay (EMSA), we reported that the core DNA‐target site of Ml proteins from *M. loti* and MucR from *B. abortus* is constituted by an AT‐rich sequence containing a T‐A step and we demonstrated that these proteins contact DNA mostly in the minor groove 17. The T‐A step is an element of DNA responsible for protein–DNA shape readout recognition 20, 21, 22 because of its flexibility, which makes the minor groove wider than it is in stiffer A‐tracts (defined as four or more succeeding adenines), which narrow the minor groove 21, 22. Furthermore, we showed that the Ml proteins and MucR form higher‐order oligomers 17. The ability to bind AT‐rich DNA‐target sites containing a T‐A step and to oligomerize forming higher‐order oligomers is also distinctive features of H‐NS, which are structuring nucleoid‐associated proteins 20, 23, 24, 25, 26, 27, 28. The H‐NS are heat‐stable proteins with a temperature of melting (*T*_m_) of 40 °C 29.

Here we demonstrate that MucR from *B. abortus* binds more than one target site in its own gene promoter. These data confirm that the highest affinity DNA‐target site of MucR is constituted by an AT‐rich sequence containing a T‐A step and suggest how this protein represses its own gene expression. Furthermore, we report a circular dichroism analysis and a thermal unfolding/refolding of MucR, demonstrating that it is a heat‐stable protein. Taken together, the results shown in this study suggest that MucR adopts a molecular mechanism to bind DNA and to regulate gene expression similar to that used by H‐NS proteins, which repress virulence genes in *Escherichia coli*,* Salmonella*,* Pseudomonas* and many other species of bacteria 30, 31, 32, 33. Finally, we provide a completely new way to interpret how *Brucella* regulates virulence gene expression and suggest a role for the prokaryotic zinc‐finger proteins of Ros/MucR family as H‐NS‐like proteins.

## Materials and methods

### Cloning, protein expression and purification

The gene encoding the full‐length MucR was cloned in pET‐22b(+) vector [MucR‐pet22(+)] as previously reported 17. MucR‐pet22(+) expression vector was used to transform *E. coli* BL21DE3. Transformed colonies were inoculated in Luria–Bertani medium and grown until OD_600_ equalled 0.45. At this point of the *E. coli* growth, the expression of the protein was induced with the addition of IPTG at a final concentration of 1 mm at 28 °C for 1 h. The purification of MucR was obtained as previously reported 17. The protein eluted from a Mono S HR 5/5 cation exchange chromatography column in the 0.4–0.8 m NaCl concentration range.

### Electrophoretic mobility shift assay

**Figure 1:**
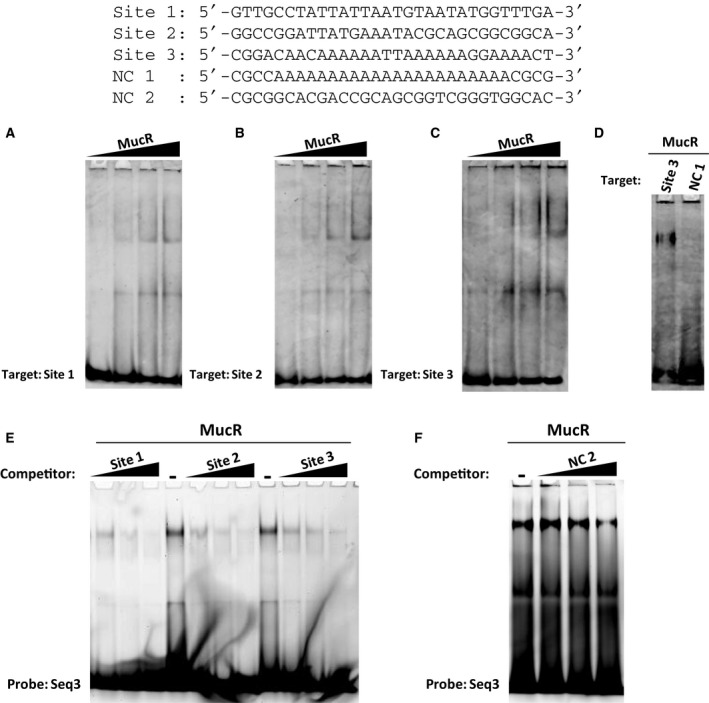
MucR binds multiple sequences in its own promoter. EMSAs of MucR from *Brucella abortus* with Site 1 (A); Site 2 (B); Site 3 (C); Site 3 and NC 1 (D). The sequences of the oligonucleotides tested as target sites are indicated. The growing amount of protein is indicated on the top of the lanes (0.5, 1.0, 1.5 and 2 μg). In the panel D, the amount of protein tested is 2 μg. (E, F) Competition assays: the growing amount of competitors (1×, 2×, 3× respect to the amount of probe) is indicated on the top of the lanes. In each lane, 1.5 μg of protein was used. The sequences of the oligonucleotides used as competitors are indicated at the top of the figure. The sequence of the probe Seq3 is reported in the Materials and methods.

The EMSA experiments were performed as previously described 17, 34. In detail, a protein amount of 0.5, 1, 1.5 or 2.0 μg were incubated 10 min on ice with 5 pmol of the double‐stranded oligonucleotides, Site 1, Site 2, Site 3 (sequences shown in Fig. 1) in binding buffer (25 mm HEPES pH 7.9, 50 mm KCl, 6.25 mm MgCl2, 5% glycerol). The total volume of the reaction mixtures was 20 μL. For panel D, a protein amount of 2.0 μg was incubated 10 min on ice with Site 3 or with NC 1 double‐stranded oligonucleotides (sequences shown in Fig. 1) in binding buffer (the total volume of the reaction mixtures was 20 μL). After incubation on ice, the samples were loaded onto a 5% polyacrylamide gel in 0.5X TBE and run at room temperature for 70 min at 200 V. Gels were stained 20 min with Diamond™ Nucleic Acid Dye (Promega) and imaged by Typhoon Trio+ scanner (GE Healthcare).

The competition EMSA experiments were conducted as previously reported 17, 35. About 1.5 μg of the protein was incubated 10 min on ice with the FAM‐labelled double‐stranded Seq3 oligonucleotide (5′‐ATTGACTAAATAATAAGCTA‐3′) in binding buffer (the total volume of the reaction mixture was 20 μL). Then, the same amount of unlabelled competitor oligonucleotides as FAM‐labelled Seq3 or a twofold or threefold excess of competitors was added to the reaction mixture (the sequences of competitors are shown in Fig. 1). After 10 min of incubation on ice from the addition of competitors, the samples were loaded onto a 5% polyacrylamide gel and run at room temperature in 0,5X TBE (200 V for 70 min). The polyacrylamide gels were imaged by Typhoon Trio+ scanner (GE Healthcare).

### CD spectra

The CD spectra and the thermal denaturation were registered as already described in Pirone *et al*. 36.

In detail, CD spectra of MucR spectra were recorded with a J‐810 spectropolarimeter equipped with a Peltier temperature control system (Model PTC‐423‐S, Jasco Europe, Cremella, LC, Italy). Far‐ultraviolet (Far‐UV) measurements (195–260 nm) were carried out in 20 mm NaP pH 7.0, 50 mm NaCl at 20 °C using a 0.1 cm optical path length cell using 5 μm of MucR. Spectra were recorded with a time constant of 4 s, a 1 nm bandwidth and a scan rate of 10 nm min^−1^. Thermal denaturation curve was recorded over the 20–100 °C temperature interval monitoring the CD signal at 222 nm at a scan rate of 1.0 °C·min^−1^.

The signal was averaged over at least three scans and baseline corrected by subtraction of a buffer spectrum. The CD spectra were repeated in three independent experiments. Deconvolutions of CD spectra were obtained using the Web‐based program CDPRO http://sites.bmb.colostate.edu/sreeram/CDPro/.

## Results

Analysing the promoter sequence of the *mucR* gene in *B. abortus* genome and the sequence of many regions recognized by MucR from *B. abortus* 4, we noticed that multiple AT‐rich sites containing T‐A steps were present and spaced by dozens of nucleotides. Based on previously published results demonstrating that AT‐rich DNA sequences are target sites of MucR 17, we focused our attention on the AT‐rich regions in the *mucR* gene promoter and we identified three AT‐rich sequences containing at least one T‐A step as putative target sites of MucR. We designed three double‐stranded oligonucleotides each containing one of the putative MucR target sites identified in the *mucR* gene promoter and we named them Site 1, Site 2 and Site 3. Site 1, Site 2 and Site 3 are located, respectively, at −174, −91 and −31 bp from the ATG start codon of *mucR* gene. To analyse the ability of MucR from *B. abortus* to bind these three sequences, we tested them by EMSA as DNA‐target sites for the purified MucR protein (Fig. 1A–C). The results show that MucR is able to bind all the three sequences tested. Interestingly, the double‐stranded oligonucleotide NC 1, with the same percentage of adenines and thymines as Site 1 and Site 3 (73% AT‐rich – Fig. 1) but with a completely different arrangement of these bases not including T‐A step, is not recognized by MucR in EMSA experiments under the conditions tested (Fig. 1D). These data demonstrate that more than one target site for MucR is available in the promoter of its own gene and confirms, as previously reported 17, that AT‐rich sequences containing a T‐A step are the preferred DNA‐target sites of MucR. To investigate the affinity of MucR for the oligonucleotides Site 1, Site 2 and Site 3, we performed a competition assay using these three oligonucleotides as competitors of MucR binding to a previously published DNA‐target site 17, the oligonucleotide Seq3 (Fig. 1D). The results reveal that MucR binds Site 1 with a slight higher affinity than Site 2, whereas the binding affinity to Site 3 decreases compared to Site 1 and Site 2. As a negative control of competition assays, we designed a GC‐rich double‐stranded oligonucleotide, named NC 2, and tested it as competitor of MucR binding to Seq 3 (Fig. 1F). The result clearly shows that NC 2 does not displace MucR binding to Seq 3 as Site 1, Site 2 and Site 3. Analysing the sequence of Site 1, we noticed that it is more AT‐rich than Site 2 (Site 1 is 73% AT‐rich, Site 2 is 40% AT‐rich), whereas it has the same adenine and thymine percentage as Site 3 (Site 3 is 73% AT‐rich as well as Site 1). Nevertheless, adenines and thymines do not form A‐tracts in the oligonucleotides Site 1 and Site 2, whereas in the Site 3 three A‐tracts are present. The A‐tracts narrow the minor groove of DNA, making it less suitable to accommodate the DNA‐binding domain of proteins which contact DNA preferentially in the minor groove 20, 21, 22 such as MucR 17. This observation leads us to hypothesize that the AT‐richness, and above all, the absence of A‐tracts could be the cause of the highest affinity of MucR for Site 1. Between the two oligonucleotides Site 2 and Site 3, Site 3 contains a higher percentage of adenines and thymines (Site 3 is 73% AT‐rich, Site 2 is 40% AT‐rich), but in this oligonucleotide, more than one A‐tract is present; this latter adenines and thymines organization disadvantages contact with the minor groove, and it could be the cause of the lower DNA‐binding affinity of MucR to Site 3 than Site 2. Taken together, the binding results indicate that the affinity of MucR for DNA is affected not only by the AT‐richness, but also by the arrangement of adenines and thymines, which favour MucR binding when they form T‐A steps, whereas disadvantage MucR binding when they form A‐tracts.

**Figure 2:**
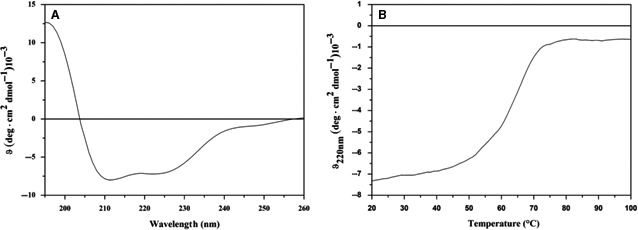
MucR is a heat‐stable protein. Far‐UV CD spectrum of MucR recorded at 20 °C (A) and thermal denaturation curve obtained by monitoring the CD signal at 220 nm (B).

Finally, we investigated the folding state and the thermal stability of MucR performing a structural characterization by Far‐UV CD spectroscopy. In accordance with CD spectra registered for H‐NS 29, the Far‐UV CD spectrum of MucR is characterized by the presence of two minima, at 208 and 222 nm, and one maximum, at 195 nm, typical fingerprints of α/β proteins (Fig. 2A). This is also in agreement with deconvolution data carried out by CD PRO performed on three independent experiments (35 ± 3% of α‐helices and 17 ± 4% of β‐sheets). The stability of MucR was also evaluated by performing a thermal denaturation analysis, monitoring the CD signal at 220 nm (Fig. 2B). MucR showed a remarkable stability with Tm values of 63 °C, higher than that observed for H‐NS (about 40 °C) 29, but, however, in line with the peculiar stable feature of H‐NS proteins 29. The unfolding process resulted to be reversible, and the sigmoidal shape of the denaturation curve indicates a monophasic helix–coil transition of the protein as already observed for H‐NS 29, suggesting the absence of stable intermediates.

## Discussion

In this paper, we demonstrate for the first time that MucR is able to recognize more than one DNA‐target site in the promoter of its own gene confirming that AT‐rich sequences containing a T‐A step are the preferred target sites of MucR. We also show that among the three DNA‐target sites tested, MucR binds with higher affinity the Site 1 oligonucleotide having a high percentage of adenines and thymines which does not form A‐tracts. Our data indicate which elements in the DNA‐target sites could be responsible for the highest MucR DNA‐binding affinity among different AT‐rich DNA‐target sites. In fact, DNA‐binding experiments suggest that not only the AT‐richness but also the arrangement of adenines and thymines affect MucR DNA‐binding affinity: the presence of adenines and thymines favours MucR DNA‐binding when they form T‐A steps, but they disadvantage binding when they form A‐tracts. A possible explanation of these results is that A‐tracts are known to narrow the DNA minor groove 20, 21, 22 whose interaction with MucR has been proven to have a key role in this protein–DNA‐binding mechanism 17.

The sequences that we have identified as MucR DNA‐target sites, located in the *mucR* gene promoter, are spaced each other by 30–50 bp. The analysis of the sequences of the gene promoters regulated by MucR 4 reveals similar organization as the *mucR* promoter with AT‐rich sequences containing a T‐A step spaced by dozens of nucleotides. We have previously reported that MucR can form higher‐order oligomers analysing its oligomeric state by light scattering and dynamic light scattering 17. The nucleoid‐associated proteins H‐NS are able to recognize AT‐rich DNA‐target sites containing T‐A steps 20, 23, 24 and to oligomerize 25, 26, 27, 28. The AT‐rich target sites are used by H‐NS as nucleation sites, which are the first bound by H‐NS; after the recognition of these AT‐rich nucleation sites, the H‐NS extend their presence on the nucleoid by oligomerizing and being able, in this way, to bind also lower affinity DNA‐target sites 23, 28, 30, 37, 38. The way that MucR uses to interact with DNA appears similar to that adopted by H‐NS, which bind with higher affinity AT‐rich target sites not containing A‐tracts 20. In fact, in this study, we have shown that the protein MucR recognizes AT‐rich DNA‐target sites with different affinities based on adenine and thymine arrangements: the DNA‐binding affinity of MucR in our EMSA experiments is higher for Site 1 than Site 2 and Site 3. Site 1 is the most AT‐rich target site tested not containing A‐tracts. Considering that the three DNA‐target sequences of MucR that we have identified are spaced by dozens of nucleotides, it seems reasonable to hypothesize that MucR represses the expression of its own gene interacting first with the highest affinity target sites present in the promoter and subsequently, through oligomerization, binding the lowest affinity target sites and extending its presence on the nucleoid as the H‐NS are able to do. However, we cannot exclude that other factors in *Brucella* can be able to bind the same sequences recognized by MucR affecting, in this way, the affinity of MucR for the target sites used in our EMSA experiments.

Furthermore, we have demonstrated that the temperature of melting of MucR is 63 °C, indicating that it is a heat‐stable protein. Also this feature of MucR is in common with H‐NS, which are defined as heat‐stable nucleoid structuring proteins 30 with a high temperature of melting 29.

Virulence genes are often acquired by horizontal gene transfer (HGT), and they are characterized by a high percentage of adenines and thymines 39, 40, 41, 42, 43, 44. The acquisition of pathogenicity islands by HGT is well described as an advantage for bacteria, which can repress virulence genes by histone‐like proteins as H‐NS and allow their expression only when the infection is possible 24, 39, 42, 43, 44. Nevertheless, in the pathogen α‐proteobacteria such as *A. tumefaciens* and *Brucella* spp. where virulence AT‐rich regions are present and whose expression is regulated by MucR/Ros family members, no H‐NS homologue has ever been identified 45. H‐NS‐like proteins can be divergent in their primary structure even if they share a common molecular mechanism to obtain gene expression regulation 45 and many nucleoid‐associated proteins are catalogued on the basis of their ability to bind AT‐rich DNA, their basic nature and their low molecular masses 46. Adopting a way to bind DNA resembling that of H‐NS proteins, sharing the basic nature and the low molecular masses (about 15 kDa), MucR from *Brucella* and the other members of Ros/MucR protein family could assume in the α‐proteobacteria the role that H‐NS play in many different species of β‐ and γ‐proteobacteria such as *E. coli*,* Salmonella typhimurium*,* Pseudomonas aeruginosa, Shigella flexneri, Vibrio cholerae* 30, 31, 32, 33, 37, 45, 46, 47. Further studies of the MucR DNA‐binding ability to the promoter regions of genes involved in the infection process from *Brucella* and studies aimed to demonstrate the ability of MucR to structure the nucleoid will be necessary to definitively confirm our hypothesis.

Our data reported in this paper, by showing that MucR can bind AT‐rich sequences spaced by dozens of base pairs and suggesting a cooperative binding of MucR to the regulatory regions present in the nucleoid, open a new way to interpret the molecular mechanism, which is adopted by the members of Ros/MucR protein family to regulate virulence gene expression. In particular for *Brucella* spp. in which *mucR* gene mutations lead to attenuated strains 3, 4, 5, the right interpretation of the molecular mechanism adopted by MucR to regulate the expression of genes involved in the infection process, has a considerable interest in an attempt to develop new strategies for vaccination against brucellosis. Our findings represent the starting point to get deeper insights into the hypothesis that the members of Ros/MucR family are H‐NS‐like proteins in the α‐proteobacteria.

## Author contribution

IB conceived and designed the project, expressed and purified MucR, performed EMSAs, analysed results and wrote the manuscript. LP and EMP performed and analysed CD spectra. GM and RF contributed to interpret structural data. EMP, PVP and RMR 2nd contributed to write manuscript. All the authors revised the manuscript.
